# Healthy families: study protocol for a randomized controlled trial of a screening, brief intervention, and referral to treatment intervention for caregivers to reduce secondhand smoke exposure among pediatric emergency patients

**DOI:** 10.1186/s12889-017-4278-8

**Published:** 2017-05-02

**Authors:** E. Melinda Mahabee-Gittens, Robert T. Ammerman, Jane C. Khoury, Lara Stone, Gabe T. Meyers, John K. Witry, Ashley L. Merianos, Tierney F. Mancuso, Kristin M. W. Stackpole, Berkeley L. Bennett, Laura Akers, Judith S. Gordon

**Affiliations:** 1 Cincinnati Children’s Hospital Medical Center and Department of Pediatrics, University of Cincinnati, 3333 Burnet Avenue, Cincinnati, OH 45229-3039 USA; 2 Division of Pediatric Emergency Medicine, Cincinnati, Ohio USA; 3 Division of Behavioral Medicine & Clinical Psychology, Cincinnati, Ohio USA; 4 Division of Biostatistics and Epidemiology, Cincinnati, Ohio USA; 5 School of Human Services, University of Cincinnati, PO Box 210002, Cincinnati, OH 45221 USA; 6 Pediatric Residency Training Program, Cincinnati, Ohio USA; 7 Center for Better Health and Nutrition (HealthWorks!), Cincinnati, Ohio USA; 8 Oregon Research Institute, 1776 Millrace Drive, Eugene, Oregon, 97403 USA; 9 College of Nursing University of Arizona, 1305 N. Martin Avenue, Tucson, AZ 85721 USA

**Keywords:** Smoking cessation, Tobacco, Pediatrics, Emergency department, Urgent care, Intervention, Parents/education, Child

## Abstract

**Background:**

Involuntary exposure to secondhand smoke (SHSe) is an important cause of morbidity in children who present to the pediatric emergency department (PED) and urgent care (UC). SHSe interventions delivered in the PED and UC would benefit both the smoker and child, but there have been no large trials testing the efficacy of such interventions. The Healthy Families program is the first randomized controlled trial to test whether a screening, brief intervention, and referral to treatment (SBIRT) intervention delivered in the PED and UC will be effective in decreasing SHSe in children and increasing cessation in smokers.

**Methods/design:**

This trial uses a randomized, two-group design in which caregiver-smokers of children 0–17 years old are recruited from the PED and UC. Eligible caregiver-smokers are randomized to either the: 1) SBIRT Condition with face-to-face, tailored counseling that focuses on the child’s illness, the importance of reducing child SHSe, caregiver smoking cessation, and the option to receive nicotine replacement therapy; or 2) Healthy Habits Control Condition which includes face-to-face, tailored attention control “5–2–1-0” counseling that focuses on improving the child’s health. Dyadic assessments are conducted in-person at baseline, and via email, phone, or in-person at 6-weeks and 6-months. The primary outcomes are biochemically-verified, 7-day point prevalence and prolonged smoking abstinence. Secondary outcomes are cigarettes smoked per week, 24 h quit attempts, and biochemically validated child SHSe at each time point. The costs of this intervention will also be analyzed.

**Discussion:**

This study will test an innovative, multilevel intervention designed to reduce child SHSe and increase smoking cessation in caregivers. If effective and routinely used, this SBIRT model could reach at least one million smokers a year in the U.S., resulting in significant reductions in caregivers’ tobacco use, SHSe-related pediatric illness, and healthcare costs in this population of children.

**Trial registration:**

ClinicalTrials.gov Identifier: NCT02531594. Date of registration: August 4, 2015.

**Electronic supplementary material:**

The online version of this article (doi:10.1186/s12889-017-4278-8) contains supplementary material, which is available to authorized users.

## Background

Involuntary exposure to secondhand smoke (SHSe) from caregivers who smoke is an important cause of morbidity and mortality in children [1]. Annual healthcare costs associated with SHSe and adult smoking are over $170 billion and increasing [2, 3]. Our research has found that among children who visit the emergency settings of the Pediatric Emergency Department (PED) and Urgent Care (UC), there is high prevalence of caregivers who smoke (up to 48%) and children of these caregivers have high levels of SHSe that may be equivalent to that of active smokers [4, 5]. The majority of these smokers have incomes at or below the poverty level, limited access to cessation resources, and frequently use the PED as a “safety net” for non-urgent, SHSe-related complaints such as colds and ear infections [4, 6–9]. In 2010, 48.2% of the 23.7 million emergency department (ED) visits for children were triaged in the non-urgent or semi-urgent category and these rates were highest in those who were uninsured or Medicaid recipients [10, 11]. These children and their caregivers are at risk for a variety of tobacco-related disparities including: increased tobacco use, reduced cessation rates and access to cessation resources, and increased adult and pediatric tobacco/SHSe-related morbidity [12–16]. Encouragingly, these caregivers are motivated to quit and eager to receive smoking cessation interventions in the emergency setting [8, 17–19].

Annually, more than three million PED visits involve treatment of children with SHSe-related illnesses [11]. These visits provide a unique “teachable moment” in which healthcare providers can leverage the caregiver’s desire to quit and the child’s current SHSe-related illness [20–22]. Moreover, the emergency setting is an ideal venue for intervention as the environment already entails long natural wait times for non-acute care, during which we and others have shown that even large, complex clinical trials can be successfully conducted without disrupting clinical flow [5, 18, 23–25]. Although tobacco cessation research has been conducted in the adult ED [26–28], large controlled trials of interventions for adult caregivers in the pediatric emergency setting have not been conducted. The delivery of cessation interventions to adults in the pediatric emergency setting would represent an evolution in the use of this setting to improve the health of both caregivers and their children. The results of such work would move the field forward, as the provision of tobacco interventions for the caregiver, *who is not the patient*, is quite different than for an *adult patient*.

The Healthy Families project is the first randomized controlled trial to test whether an innovative, multilevel screening, brief intervention, and referral to treatment (SBIRT) intervention delivered in the PED and UC will be effective in decreasing SHSe in children and increasing cessation in smokers compared to an attention control condition (Healthy Habits Control, HHC). In addition, a cost analysis will be conducted to assess the costs and cost savings of this intervention.

### Aims and hypotheses

Primary Aim 1: To evaluate the efficacy of the SBIRT condition compared to the HHC attention control condition on caregiver smoking.

Primary Aim 2: To evaluate the efficacy of the SBIRT condition compared to the HHC condition on reducing children’s SHSe.

Primary Aim 3: To explore mediators and moderators of the SBIRT outcomes.

Hypotheses are:

1. Caregivers who receive the SBIRT will have higher prolonged abstinence and point prevalence cessation rates compared to caregivers in the HHC condition.
2. Children of SBIRT caregivers will have higher total home/car smoking bans and lower home/car smoking exposure compared to children of HHC caregivers.
3. The SBIRT intervention effect on prolonged abstinence and point prevalence cessation rates will be mediated by the caregiver’s perception of the risk of smoking on their child’s health and the caregiver’s motivation to quit over time. The intervention effect will be moderated and attenuated by factors such as household smokers and financial strain.

Secondary Aim 1: To conduct a cost-effectiveness analysis of the intervention from the organizational perspective, including collecting data on costs incurred and cost savings resulting from the intervention.

## Methods/design

### Overview of study design

This study is a RCT with 750 caregivers who smoke and their children who are recruited during the child’s PED or UC visit. Participants are randomized to one of two conditions: (1) SBIRT Condition with a face-to-face, tailored counseling intervention that focuses on the child’s illness and the importance of reducing child SHSe by helping caregivers quit smoking and the option to receive nicotine replacement therapy (NRT); or (2) HHC Condition which includes a tailored, face-to-face attention control intervention that focuses on improving the child’s health by encouraging the “Let’s Go! 5–2–1-0” health practices to prevent youth obesity [29, 30].

The SBIRT condition will use components shown to be effective in the outpatient setting but not yet tested in the pediatric emergency setting. It will include: 1) an abbreviated form of the Clinical Practice Guideline: Treating Tobacco Use and Dependence [31], 2) individualized, tailored, motivational interviewing (MI), 3) written materials on quitting smoking and SHSe, 4) immediate access to caregivers’ choice of cessation resources (e.g., Tobacco Quitline (QL), smokefree.gov, or smokefreeTXT), 5) a 6-week supply of NRT at baseline, 6) weekly booster messages for 12 weeks, and 7) a supplemental NRT supply at 6-weeks. The HHC program has been previously developed and used in the outpatient setting, and will be used as an attention control condition which will mirror the SBIRT condition in which caregivers will receive instruction on healthy lifestyle choices [29, 30] to prevent childhood obesity.

Electronic self-report assessments are conducted in-person at baseline (t_0_) and via email, phone, or in-person during home visits at 6-weeks (t_1_) and 6-months follow-up (t_2_). The primary outcomes for caregivers are self-reported 7-day point prevalence and prolonged abstinence from tobacco at t_1_ and t_2_, validated in all participants via exhaled carbon monoxide. Secondary outcomes for caregivers are reported: reduction in cigarettes smoked, 24 h quit attempts, readiness to quit, and use of cessation resources. Child outcomes are caregiver reports of total home/car smoking bans and reductions in SHSe, validated using salivary cotinine in all children at t_1_ and t_2_. The schedule of enrollment, interventions, and assessments is illustrated in Table 1. Participants are recruited over 48 months. Participants are allocated into either the SBIRT or HHC, using a stratified randomization scheme, after initial baseline assessment at t_0_. Our trial design follows the SPIRIT checklist for standard protocol items [32, 33] (see Table 1 and Additional file 1).

**Table 1:** Schedule of enrollment, interventions, and assessments

	Study Period			
	Enrollment	Allocation	Post-allocation	
Time Point	Baseline t_0_	Baseline t_0_	6 weeks t_1_	6 months t_2_
Enrollment				
Eligibility Screen	X			
Informed Consent/Assent	X			
Allocation		X		
Interventions				
Intervention Group (SBIRT)		X		
Control Group (HHC)		X		
Assessments				
Sociodemographics	X	X		
Stages of Change		X		
Nicotine dependence, motivation to quit, quit attempts		X	X	X
Child SHSe		X	X	X
Covariates, mediators, moderators of the intervention		X	X	X
5–2–1-0 Behaviors		X	X	X
Use of NRT and cessation resources			X	X
Child SHSe-related healthcare visits		X	X	X
Self-reported child SHSe			X	X
Biochemically validated quitting outcomes			X	X
Biochemically validated SHSe outcomes			X	X

### Participant inclusion/exclusion criteria

Adult participants are required to meet the following criteria in order to be eligible for enrollment in the trial:

1. Age 18 or older
2. Parent or legal guardian of a child 0–17 years of age who is presenting to the PED or UC with a: (a) “non-urgent” or “urgent” triage category and (b) potentially SHSe-related chief complaint (such as wheezing, difficulty breathing, cough) as outlined by the U.S. Surgeon General [1]
3. Daily smoker
4. English literate
5. Have a permanent address and a working cell or landline number

Exclusion criteria:

1. Tobacco chewers only
2. In a tobacco cessation program or on pharmacologic cessation medications
3. Live more than 50 miles away
4. Plan to move with no permanent address within 6 months of enrollment
5. Child is an active smoker
6. Child is tracheostomy dependent

### Study procedures

#### Setting

Participants are recruited from the main PED and UC of Cincinnati Children’s Hospital Medical Center (CCHMC). CCHMC is a 673-bed, freestanding, academic, pediatric medical center with more than 1.3 million patient encounters annually. The PED has over 90,000 annual visits and the UC sites have over 73,000 annual visits.

#### Recruitment and study flow

Trained clinical research coordinators (CRC) screen consecutive parents/legal guardians of patients presenting to the PED or UC using CCHMC’s electronic medical record system, Epic. CRCs will identify caregivers by reviewing Epic records of registered patients. The CRC will approach potentially eligible caregivers to screen for eligibility. The study will be explained to eligible caregivers and informed consent will be obtained by the CRC; children over age 11 will provide assent. A secure database will be used during enrollment times, which will include all patients approached, their age, sex, race, chief complaint, enrollment status, and reasons for non-enrollment (if applicable).

#### Randomization

An electronic stratified random block randomization scheme will be compiled by the study biostatistician prior to the start of the study, and will be used by the CRC to allocate participants to each condition after participants complete the baseline assessment, which includes stage of readiness to quit smoking [34]. The biostatistician has a SAS® program for creating the randomization scheme that has been tested and used for other studies. Randomization is stratified by sex of the primary caregiver and stage of readiness to quit smoking where participants will be in stages: 1) Pre-contemplation; 2) Contemplation 1 (i.e. not planning to quit in the next 30 days and had a prior quit attempt in the past year); 3) Contemplation 2 (i.e., planning to quit in the next 30 days and did not have a prior quit attempt in the past year); or 4) Preparation [35–37]; thus there will be eight strata for randomization. As it is anticipated that there will be fewer male caregivers, the random block sizes used will be smaller than for the females. The randomization scheme will be accessed by the CRC by logging into a dedicated website within Research Electronic Data Capture (REDCap) [38] and entering the sex and readiness to quit stage; the study group assignment and a study identification number will be returned. Backup will be opaque envelopes, kept within the PED with stratification and study identification number marked on the outside. The “envelope” randomization information will be entered into the website by the CRC, and the envelopes used will be retained. Participants are informed of their study assignment at baseline upon completion of randomization.

### Treatment conditions

#### Screening, intervention, referral to treatment (SBIRT) condition

##### Theoretical Framework of the SBIRT Intervention

The SBIRT is based on three premises. 1) Because our prior research demonstrates that over 50% of the caregivers who smoke will be low income [7, 8, 18], the SBIRT must target this population. Accordingly, the SBIRT is clear, brief, engaging, and easy to understand. The content of the SBIRT will be framed around perceived child and caregiver risk of smoking and motivation to change as these have been found to be very impactful in achieving cessation in this population in other settings [39–42]. Moreover, follow-ups are provided in the caregiver’s home and on a schedule that considers the logistical challenges faced by our target population. 2) Applying the SBIRT when the child is seen in a medical setting provides a “teachable moment” to potentiate a behavior change such as quitting. 3) Barriers and motivators to quitting are crucial proximal determinants of continued tobacco use and these are targeted in the SBIRT. The theoretical model guiding SBIRT is the social cognitive theory [43–45]. This theory stipulates that beliefs, attitudes, prior experiences, and contextual determinants drive and maintain tobacco use and contribute to addiction. The theoretical foundation of the SBIRT is also informed by more circumscribed but relevant perspectives, e.g., the precaution adoption model [46–48] and the transtheoretical model’s stages of change [35–37].

**Fig. 1:**
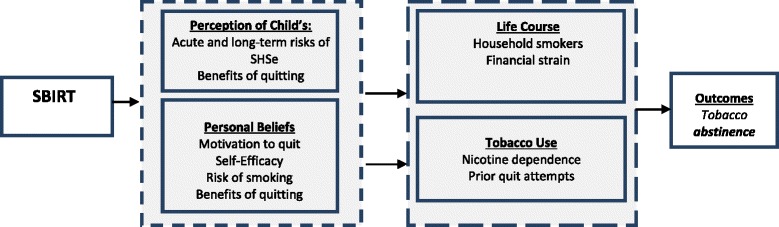
Theoretical Framework of the SBIRT Intervention

This integrated model, which guides this research and reflects the proposed mechanisms of action for the SBIRT, is presented in Fig. 1. The SBIRT takes into account the unique needs, circumstances, and resources of the population that is the focus of the study. We assert that the SBIRT will lead caregivers to leverage and problem-solve proximal determinants or precursors of cessation. The primary targets for the SBIRT are the caregiver’s perceptions about their child’s risk of SHSe, benefits to the child of quitting, and beliefs about quitting. The SBIRT will amplify these issues in the context of the child being brought in for treatment, thereby permitting a discussion about the risks of continued smoking and the benefits of quitting. It will work on heightening the caregiver’s innate self-concept and altruistic role as a provider for their child who wants to help improve their child’s health [49–51]. Life course and tobacco use variables strongly moderate smoking outcomes, and contribute to the content of the 10–15 min SBIRT session when barriers and facilitators to quitting emerge. By targeting caregiver’s beliefs and incorporating barriers and facilitators to quit in this unique setting, the likelihood of quitting and sustained abstinence is substantially increased.

#### Components of the SBIRT Condition

The trained SBIRT interventionist (see Section on Interventionist Training, below) will be introduced to participants randomized to the SBIRT condition by the CRC or Principal Investigator (PI). These caregivers will receive an individualized, brief (10–15 min) “Advise, Assess, Assist/Arrange” intervention based on the Clinical Practice Guidelines, and tailored on levels of motivation to quit, tobacco dependence, and the child’s current illness [31]. If both caregivers are present, eligible, and wish to receive the intervention, then the interventionist will provide each caregiver with the “Advise” portion of the intervention. However, only the primary caregiver (designated by the couple) will be enrolled in the study and assessed for data collection. The intervention components that will be provided are as follows:

##### Advise

The interventionist will discuss the effects of SHSe on their child’s health, the benefits of quitting, and how quitting could improve their child’s current medical symptoms and decrease future PED visits. MI techniques will be used to increase caregiver’s risk perceptions of his/her child’s vulnerability to SHSe and the health risks associated with smoking to the smoker and to the child [39–42, 52]. The interventionist will elicit the caregiver’s beliefs about his/her tobacco use, the importance he/she places on quitting and the confidence he/she has to quit. The interventionist will ask about specific barriers and facilitators to quitting (e.g., financial stress, other household smokers, prior quit attempts, and rewards associated with quitting). They will collaborate to increase confidence in quitting, problem-solve how to bypass barriers, and create action steps to quit. All caregivers will be encouraged to implement total smoking bans as a way to decrease SHSe [42, 53, 54]. For those not wanting to quit, options will be discussed, and goals set for reducing SHSe (e.g., gradually increasing the proportion of cigarettes smoked outdoors up to eliminating indoor/car smoking completely).

##### Assess

The interventionist will assess the caregiver’s readiness to quit by asking if they want to quit and are willing to set a quit date in the next 14 days. MI techniques will be used to increase readiness to quit for caregivers who do not express an immediate interest in quitting, as well as to solidify and maintain commitment in those ready to proceed.

##### Assist/Arrange

This step will consist of three components:

1) NRT: All caregivers who are willing to set a quit date within 14 days are asked if they are interested in receiving a 6-week supply of NRT (choice of patch or lozenge) during the PED or UC visit. The CRC (who will receive training on NRT) will screen all participants for eligibility and for any contraindications to NRT using a pharmacy and Institutional Review Board approved checklist. The completed checklist is either shown to, electronically sent, or read aloud to a study physician to verify eligibility. In addition, all caregivers will be provided a home visit for follow-up at 6-weeks during which they will receive an additional six weeks of NRT, if eligible.

2) Immediate connection to cessation resources: All caregivers will be provided an “Assisted Referral” based on preference. These options will include: a) QL: The interventionist will dial a direct number to the state QL and a QL counselor will initiate brief counseling. The Ohio QL provides counseling calls and may provide NRT to select participants [55]; b) Web-based: the interventionist will help the caregiver log onto smokefree.gov using the computer that is in every patient’s PED or UC room. This site has a variety of interactive tools including a LiveHelp online “chat” service, SHSe and cessation quizzes, a cessation mobile app, and other helpful resources; and c) Text Messaging: the interventionist will help the caregiver sign up with smokefreeTXT which is on smokefree.gov. These free mobile services provide encouragement, advice, and tips to help smokers quit.

3) Intervention Materials: To supplement the baseline intervention, the caregiver will be provided with free materials shown to be effective in previous studies [56, 57]. Weekly booster messages with reminders of the benefits of quitting will be sent by email, mail, or text message (caregiver’s preference) for 12 weeks.

*Components of the HHC Condition.*

Trained interventionists will deliver the HHC intervention (See Interventionist Training, below). Training, intervention orientation, and intervention/counseling procedures will mirror those of the SBIRT condition, but focus on the childhood obesity prevention program entitled “Let’s Go! 5–2–1-0”, a program which has been developed and used in the outpatient clinic and school setting. This program is designed to help children and families eat healthy and be active, by recommending the following daily behaviors: five fruits and vegetables, two hours or less of screen time, one hour or more of physical activity, and zero sugary drinks [29, 30]. The HHC intervention will be tailored on current diet, screen time, and activity levels. If both caregivers are present, eligible, and wish to receive the intervention, then the interventionist will provide each caregiver with the intervention. However, only the primary caregiver (designated by the couple) will be enrolled in the study and assessed for data collection. The intervention components that will be provided are:

##### Advise

Caregivers will be advised by the interventionist about helping their children and families to eat healthily and be active. He/she will give them recommendations on daily nutritional and healthy lifestyle guidelines with the 5–2–1-0 behaviors. MI techniques will be used to increase the caregiver’s risk perceptions of poor diet and activity choices and the interventionist will ask about specific barriers and facilitators to making and adhering to these choices.

##### Assess

The interventionist will assess caregivers’ readiness to make good lifestyle choices by asking which area of the 5–2–1-0 recommendations they want to improve in the next 14 days. MI techniques will be used to increase readiness to change habits for caregivers who do not express an immediate interest, as well as to solidify and maintain commitment in those ready to proceed.

##### Assist/Arrange

This step will consist of the following components:

1) Water bottles: All caregivers will be offered a colorful water bottle to encourage children to drink more water.

2) Immediate connection to 5–2–1-0 resources: All caregivers will be provided with the option to log onto and view a website with information and ideas on how to stay healthy using 5–2–1-0 such as ideas for non-screen or video game related activities and information on how to read food labels.

3) Intervention Materials: To supplement the baseline intervention, the caregiver will be provided with free materials used in other healthcare settings (e.g., 5–2–1-0 brochures, 5–2–1-0 assessment questionnaires, “Fun ways to be physically active” pamphlets, and “Nonfood Rewards at home” pamphlets) which are downloadable from www.letsgo.org [30]. They will receive a weekly email, mail, or text message (caregiver’s preference), for 12 weeks, reminding them of healthy lifestyle behaviors. They will be offered the option to receive educational information from the SBIRT intervention at the study’s conclusion.

*SBIRT and HHC Interventionist Coverage and Training.*

In order to provide coverage for both intervention arms during study hours (which vary from 9 am to 9 pm weekdays), an equal number of interventionists will be trained for the SBIRT and for the HHC condition. Interventionists will all be, at minimum, college graduates with degrees in either social work, psychology, or education. All interventionists receive formal MI training from a licensed, MI expert. This full two-day training, followed by an eight-hour booster session after four months, will teach them how MI can be used as a collaborative, person-centered form of guiding to elicit and strengthen motivation for change and will help them use specific skills related to MI.

The interventionists who deliver the SBIRT will be trained as Tobacco Treatment Specialists at an accredited Tobacco Treatment Specialist Training program. This training will teach them how to deliver an evidence-based, cognitive behavioral treatment for nicotine dependence. The PI (MMG) and Co-Investigator (JG) will then provide them with an “Assisted Referral” training session focused on study logistics, the delivery of brief cessation and SHSe reduction counseling, direct connection to the state QL, demonstration of smokefree.gov, and enrollment in smokefreeTXT during the PED or UC visit.

The interventionists who deliver the HHC will receive training from the PI (MMG) and Co-Investigators (JG and KS) on the rationale and scientific basis for the 5–2–1-0 program [29, 30], information on the purpose and importance of talking to caregivers about healthy habits to improve their child’s health, guidance on engaging and delivering the intervention to caregivers during the PED or UC visit; barrier identification and reduction techniques; and realistic simulations of cases and appropriate intervention delivery.

Additional booster training sessions for all interventionists will be conducted by the Co-Investigator (JG) and the MI expert as needed to problem solve issues that come up and to provide refreshers and reminders about the key elements of the interventions to prevent drift and maximize consistency.

### Assessment procedures

#### Measures

##### Primary and Secondary Outcome Variables

Participant self-report data are collected via electronic survey at baseline (t_0_) during the emergency visit; data is sent to a central, secure, HIPAA-compliant firewalled CCHMC database using REDCap. All databases are only accessible by the study team. The primary dependent variable – caregiver smoking cessation – will be assessed via caregiver-reported 7-day point prevalence, defined as abstinence for the seven days prior to assessment, and report of prolonged abstinence at the 6-weeks (t_1_) and 6-months (t_2_) follow-up assessments; biochemical validation will be via exhaled carbon monoxide breath testing. Secondary caregiver outcomes will include cigarettes smoked, quit attempts, readiness to quit, and use of NRT and cessation resources (QL, smokefreeTXT, smokefree.gov). The secondary dependent variable - child SHSe - will be measured in two ways. First, child cotinine will be collected in saliva samples taken at t_0_ and t_1_; if the caregiver reports abstinence at t_2_, child saliva samples will be collected to verify the presence of home/car smoking bans. Second, child SHSe will be measured by caregiver report of the number of smokers and the number of cigarettes to which the child is exposed each day - indoor, outdoors, and in the car that the child regularly rides in - during the seven days prior to all assessment periods [58, 59]. Additional secondary child SHSe-related outcomes will be presence or absence of total home/car smoking bans and number of SHSe-related emergency visits and hospitalizations at CCHMC. Additionally, caregivers will be asked about which components of the intervention they received (e.g., advice to quit smoking) and their satisfaction with the intervention.

#### Covariates, Mediators, and Moderators

Three variables will be measured as potential covariates and possible moderators of intervention effects: 1) nicotine dependence to be measured using two items in the Heavy Smoking Index [60, 61] and prior quit attempts, 2) financial strain (three items measured on 5-point Likert scales and averaged to create a financial strain score (α = .802; higher scores will reflect more financial strain) [62], and 3) number of household smokers. Sociodemographics and smoking history variables will be assessed for possible association with outcomes. We will adapt the following measures to assess potential mediators of the intervention: perceived child vulnerability (five items rated on a 4-point scale, α = .86. Higher scores indicate higher perceived vulnerability), child precaution effectiveness (five items rated on a 4-point scale; α = .94. Higher scores indicate higher perceived benefit) [63–65]; and motivation to quit smoking using the Contemplation Ladder and stages of change to quit smoking [34, 66].

#### Follow-up assessments

Outcome assessments will not be blinded if done during home visits since assessors have to be prepared to give SBIRT participants additional NRT, if eligible. At t_1_, we will conduct home visits to: 1) complete assessment of outcomes. Participants who have not completed the assessment via phone or email complete it electronically during the visit; those who have completed it verify the validity of their answers and make any changes, if necessary; 2) verify abstinence on 100% of caregivers who report that they have not smoked in the past seven days via exhaled carbon monoxide testing; 3) obtain a follow-up salivary cotinine level on 100% of children to assess SHSe and to validate total home/car smoking bans in children of caregivers who report abstinence; and 4) determine if SBIRT participants who received NRT at baseline are interested in receiving more. If so, an eligibility checklist is completed on participants who used, at minimum, approximately 80% of the baseline supply. The completed checklist is either electronically sent or read to a study physician to verify eligibility. If eligible, an additional 6-week supply is given to participant. Follow-up home visits at t_2_ will only be conducted if the caregiver reports that they have not smoked in the past seven days via phone or email survey.

#### Saliva Sample Collection

All children will have saliva collected at t_0_ and t_1_; saliva will be collected at t_2_ during home visits if caregivers report that they have not smoked in the past seven days. Cotinine is generally accepted to be the best of the available biomarkers, with over 95% specificity and sensitivity, it is noninvasive, and easily obtained in young children [67, 68]. A standard protocol will be used to collect saliva samples using an oral swab from each child and adult; cotinine analyses will be performed with Enzyme Immunoassay techniques by Salimetrics LLC, State College, PA [69]. In the child samples, the lower limit of cotinine detection is 0.15 ng/ml (range of detection: 0.05–200 ng/ml); children will be classified as exposed to tobacco smoke if their cotinine level is >0.15 ng/ml [69].

#### Retention Strategies

Multiple strategies will be used to retain the sample including: 1) Generous incentives: Participants will be paid with increasing compensation of $30 at baseline, $50 at 6-weeks ($20 for the questionnaire, $30 for the home visit), and $65 at 6-months ($30 for the questionnaire, $35 for the home visit), plus a bonus $10 if they complete both of the follow-up assessments; 2) Conducting home visits on all caregivers and children at the 6-week follow-up to assess outcomes and giving caregivers in the SBIRT group an additional 6-week supply of NRT; 3) Hiring and training CRCs to carefully track participants, make home visits, and obtain follow-up data by making as many follow-up attempts as is necessary to locate participants; 4) Sending caregivers: weekly email/mail/text booster messages with cessation or healthy habits tips for 12 weeks, and reminders of upcoming assessments. We will ask order of preference of type of reminders and follow-up; however if one technique is unsuccessful, the second and finally third choice will be used; 5): mailing postcards: immediately after the baseline visit to thank them for participation; and at four weeks and five months to remind them of upcoming study visits. They will be emailed the 6-week and 6-month assessments one month and 5.5 months after the baseline visit, respectively.

#### Process and treatment Fidelity assessments

In order to promote fidelity and uniform implementation of the SBIRT and HHC interventions, the interventionists will complete a fidelity checklist after each session. Separate checklists for each condition will include key prescriptive and proscriptive elements for each condition. Sample prescriptive elements for the SBIRT include recommending total home/car bans, asking visitors to smoke outside, using NRT and other cessation resources to quit; sample proscriptive elements are the presentation of the effects of SHSe on children, brief statistics on SHSe and child health outcomes, and the beneficial effects of quitting on children. Sample prescriptive elements for the HHC include recommending five fruits and vegetables each day, gradually decreasing screen time, and getting kids active with fun options; sample proscriptive elements are the presentation of the health effects of poor diet and inactivity.

In addition, every SBIRT and HHC intervention will be audio recorded. Interventionists will be trained in MI strategies and techniques to criterion, and a random selection of 5% of each of the interventionists’ audio-recorded sessions will be independently rated for adherence using the Motivational Interviewing Treatment Integrity system by our MI expert, who will provide individualized written and verbal feedback to each interventionist [70]. These recorded sessions will be collected in an ongoing basis to facilitate fidelity to MI. MI approaches have been successfully applied in the emergency setting for a variety of issues, including alcohol and smoking [71]. Independent of these ratings, 10% of the audio-recordings will be randomly selected and regularly reviewed by the PI to oversee the interventionists to ensure the consistent implementation of the two conditions; interventionists will receive direct feedback from the PI on these reviews.

#### Data quality control

The PI or lead CRC will coordinate and monitor all recruitment and assessment procedures. All study data of screened and enrolled participants will be identified by study ID numbers to protect the confidentiality of participants. The PI, CRCs, and investigators will have monthly meetings to review the study progress and procedures and to discuss any adverse events or dropouts; the PI and CRCs will have at least weekly meetings to discuss study procedures. A data monitoring plan, including stopping guidelines is in place. A data monitoring committee consisting of a non-study and non-sponsor related epidemiologist, environmental health researcher, and substance abuse researcher will meet annually; there is no planned interim analysis. The data collection, management, analysis, interpretation and production of publications will be independent from the funding bodies and other competing interests. The trial results will be disseminated via journal publication and conference presentation, without exposing the identity of the trial subjects.

#### Data analyses

##### Power Analysis

Based on the recommendations of Hughes et al. [72, 73], we will use two criteria for abstinence: 6-month point prevalence (defined as 7-days of abstinence), and prolonged abstinence at 6-months. Based on our prior dental health study, we expect that 6-month point prevalence in the HHC condition will be 6.3%, and prolonged abstinence rate of 2.1% in the HHC group [56]. We are basing our estimate of 6-month point prevalence of the SBIRT group on the odds ratio derived from meta-analyses assessing the effectiveness of NRT vs. a control, ranging from 1.4–4.6 [56], to give a cessation rate of 10–12% in the SBIRT group. However, since an interventionist will be conducting the intervention, we expect high compliance in implementation. Thus, we are estimating a cessation rate of 12% in the SBIRT group. Similarly, for prolonged abstinence, we are assuming a rate of 7% in the SBIRT group.

Power analyses are based on estimates of differences in both 6-month point prevalence and 6-month prolonged abstinence between groups. With 300 subjects in each group, we would have 80% power to detect an increase of 6.4% in cessation rate. However, assuming a multiple imputation approach, we would be able to detect a 5.5% increase in cessation rate. For 6-month abstinence, we can detect an increase of 4.5% with 300 subjects per group and 4.2% with the multiple imputation assumption. These estimates are consistent with the predicted cessation rates shown above and published rates [56, 74, 75]. For analyses based on regression techniques, using hierarchical linear modeling, sample sizes will be more than sufficient. The effect sizes of interest for these analyses are the correlation coefficients and squared, semi-partial correlations (squared = incremental R^2^). To predict caregiver level variables (Primary Aim 1), with a sample size of 750 and 20% lost to follow-up, we will have >80% power to detect a correlation of 0.11 (r^2^ = 0.013). For examination of smoking bans (Primary Aim 2), these samples sizes would allow us to detect a difference of 12% with 80% power with 300 per group assuming a rate of between 50% and 65% in the HHC group. Using multiple imputation and 375 per group, we could detect a difference of 11% with 80% power.

#### Primary Analyses

This is a two-group RCT, wherein caregivers are randomly assigned to the SBIRT Condition vs. HHC Condition. The appropriate statistical models for analyses of data for inclusion of time dependent outcomes are General Linear Mixed Models (GLMM) for data either with a Gaussian/normal distribution or with a binomial or Poisson distribution, using the appropriate link function, invoking Generalized Estimating Equations (GEE) to account for the longitudinal aspect of the data [76].

We will use GLMM to evaluate the relative effectiveness of the SBIRT versus HHC Condition on our primary outcomes: point prevalence and prolonged abstinence at 6-months. The analysis will be repeated with caregiver level differences between groups included as covariates. We will use mixed model analysis of covariance, with repeated measures to assess change (controlling for baseline differences between groups) to assess the effect of the SBIRT on secondary outcomes including reduction in use, number of quit attempts, and changes in readiness to quit. Kappa statistic will be used to assess agreement between the caregiver’s self-report and the assessment of tobacco use based on salivary cotinine level at 6 months. It is possible that use of and adherence to the NRT will influence outcomes in the intervention group. In addition, some of the participants in the control condition may obtain NRT during the study interval. We will examine NRT use or dose, defined as self-reported adherence, as a covariate, in order to control for this possible relationship.

##### Primary Aim 1, Hypothesis 1

We hypothesize that SBIRT participants will have higher prolonged abstinence and point prevalence cessation rates compared to those in the HHC group. As stated above, our primary approach to the analysis is using multiple imputation, with the interventionist included in the model. We shall then use analysis of covariance, to control for any baseline differences of caregivers between conditions. In addition, we will also control for motivation to quit, considered a priori as a potential covariate, using the score on the contemplation ladder. As we are stratifying on motivation to quit and caregiver sex, we will initially enter this into the model as an interaction. To address child cotinine as the 6-month outcome, we will use a t-test and then an analysis of covariance to incorporate the variables different at baseline. Incorporating the 6-week outcome values, we will use GLMM invoking GEE for the repeated measures and a model including covariates. This approach will be used to assess the intervention’s effect on abstinence and point prevalence and child cotinine.

##### Primary Aim 2, Hypothesis 2

We will evaluate the effectiveness of the SBIRT on total home/car smoking bans and on child SHSe which will be measured by both caregiver report and child salivary cotinine, as recommended by Halterman et al. [77]. The reliability of caregiver’s reports will be examined by looking at the association between amount of smoking and SHSe levels reported at 6-months using Pearson’s correlations. For validation, we will compare the child’s salivary cotinine concentrations for those reporting total home/car smoking bans and those not, we shall also examine the change in cotinine levels from baseline for those initiating bans vs. not. The computational procedure will use data from the two measurement points, controlling for the within-subject correlation of measures repeated over time [78, 79]. The main analysis will use GLMM, first to assess the intervention effect alone and then including covariates. First, we will investigate immediate intervention effects based on change from baseline to 6-weeks post-intervention. Next, we will investigate change from 6-weeks to 6-months to examine maintenance effects. To examine overall change, we will use GEE, examining linear and quadratic terms for time, group, and group × time interactions as explanatory variables [80]. Use of NRT, described above, may be of particular interest as a covariate for this analysis.

##### Primary Aim 3, Hypothesis 3

We hypothesize that the SBIRT intervention effect on prolonged abstinence and point prevalence cessation rates will be mediated by the caregiver’s perception of the risk of smoking on their child’s health, or on their readiness to quit over time [35, 81–85]. Bivariate analyses will be used to initially examine if the variables measuring this phenomena, susceptibility and benefits of quitting are associated with cessation at each time point and if there are any changes in perceived health risk from baseline to 6-months in the SBIRT versus HHC groups. We will build on the models from our primary aim to determine the association of these potential mediators. Analysis assessing these effects will be conducted using abstinence at each follow-up point as the dependent variable and testing for interactions between treatment (i.e., intervention) and the potential mediator. Additionally, we will examine the main effects of the intervention within each level of the mediator to establish whether the intervention is effective in each subgroup, acknowledging that we may be underpowered for some subgroups.

##### Mediation analysis

To assess whether our intervention improved cessation via the proposed mediators, we will perform mediation analyses using both the criteria of Baron and Kenny [86] and Holmbeck [87]. As our outcome variables are dichotomous, we will apply the method of Huang et al. [88], which involves a system of logistic regression models and the Monte Carlo method to assess posterior distributions of the direct, indirect, and relative indirect effects. Three sets of logistic regression analyses will be performed, as the variables of interest; outcome, intervention and mediator are mainly categorical. The first two steps are to assess the intervention effects on the outcome and the mediator. In the third step, we will assess the relationship between each of the proposed mediators and the study outcome at each follow-up. The same three steps will be repeated with adjustment for potential confounders and/or moderators as determined from analysis for Hypothesis 3. The mediating variables that will be considered are: perception of child’s risk from SHSe and the benefits to the child of quitting, motivation to quit, and self-efficacy. Moderating variables are household smokers, financial strain, nicotine dependence, and prior quit attempts.

In the final analysis, the entire theoretical model to predict outcome will be examined using structural equation modeling (SEM). The strength of SEM in examining the entire model is that this method allows for the analysis of a hypothesized pattern of directional and non-directional linear relationships among a set of latent variables (e.g., caregiver beliefs about their child’s SHSe risk) and measured variables (e.g., point prevalence) [89]. This approach will allow us to examine the effect of perceived risk of SHSe and perceived child benefits of quitting; we will incorporate the former as latent variables over the study period. We will also be able to examine the relative relationship of caregiver’s beliefs and readiness to quit with the outcome of cessation.

#### Secondary Analysis

##### Secondary Aim 1

We will collect the cost data needed to conduct a cost analysis of the SBIRT intervention. The cost inventory will include data collected from CRCs and interventionists in both conditions about cessation-related activities and expenses, self-reported abstinence, and use of all tobacco products at each follow-up. Cost inputs will include staff time for tobacco screening, chart flagging, cessation counseling, giving informational materials and NRT, and referral to the QL, smokefree.gov, or smokefreeTXT. To determine costs of SHSe-related PED visits and hospitalizations at CCHMC, data will be collected from hospital records for the 6-month period before and after baseline for SHSe-related illnesses such as asthma, and compiling the associated costs.

We anticipate that the main cost component for intervention delivery will be the minutes the interventionist spends in counseling. Although the intervention will occur during the time the patient is waiting for care, the time the interventionist spends represents an opportunity cost of other alternative ways of spending this time with caregivers and should be tracked for inclusion in the cost-effectiveness analysis. Because the intervention will be delivered during visits of normal length, we do not anticipate that PEDs or UCs will incur any other overhead costs (e.g., facilities) due to adoption of the intervention.

## Discussion

This is the first RCT to test the effectiveness of an emergency setting-based intervention on child SHSe reduction and caregiver smoking cessation intervention compared to an active control condition. This intervention has the potential to improve the health and reduce tobacco-related morbidity of both caregivers and children. Building upon and expanding prior successful intervention approaches from the adult emergency setting [26, 28], we will integrate personalized behavioral and pharmacologic strategies with free, disseminable cessation resources to improve cessation rates in caregivers. Since the proposed SBIRT intervention includes multilevel and synergistic cessation components, a state-of-the-art RCT design with an active attention control group, and the biochemical validation of self-report assessments, it will provide robust unbiased evidence to objectively evaluate the effects of the intervention. The proposed model is consistent with the NIH roadmap to advance the science of behavior change by testing multilevel interventions [90]. Furthermore, the proposed project also represents a transformation in the use of the pediatric emergency visit to conduct theoretically-grounded research in this unique setting that can be used to target and improve the health of both adult smokers and children. The results of this study will improve our understanding of how to conduct and interpret the results of adult tobacco cessation research in the pediatric emergency setting. The knowledge gained may have applications in other adult behavioral interventions in this setting. The results may be applicable to other interventions (e.g., alcohol and drug use, domestic violence, and HIV testing and treatment) in which adults who bring their children to the emergency setting are screened and counseled [91, 92].

If effective and routinely used, the SBIRT model could reach at least one million smokers a year in the U.S., resulting in significant reductions in caregivers’ tobacco use, SHSe-related pediatric illness, and healthcare costs in this population. If successful, we will create a comprehensive package of implementation materials, and disseminate the intervention throughout child emergency and urgent care settings nationwide.

## Additional file

Additional file 1: SPIRIT 2013 Checklist: Recommended items to address in a clinical trial protocoland related documents. (DOC 122 kb)

## Abbreviations

CCHMC: Cincinnati Children’s Hospital Medical Center
CRC: clinical research coordinator
ED: emergency department
HHC: Healthy Habit Control
MI: Motivational Interviewing
NRT: nicotine replacement therapy
PED: pediatric emergency department
PI: Principal Investigator
QL: Quitline
RCT: randomized controlled trial
SBIRT: Screening, Brief Intervention, and Referral to Treatment
SHSe: secondhand smoke exposure
UC: urgent care
